# Rowell syndrome following tumor necrosis factor-α (TNF-α) inhibitor therapy

**DOI:** 10.1093/omcr/omaf066

**Published:** 2025-06-27

**Authors:** Robin Sia, Maree Micallef

**Affiliations:** Department of Rheumatology, Northern Hospital, Epping 3076, Victoria, Australia; Department of Rheumatology, Royal Melbourne Hospital, Parkville 3052, Victoria, Australia; Department of Rheumatology, Royal Melbourne Hospital, Parkville 3052, Victoria, Australia

**Figure 1:**
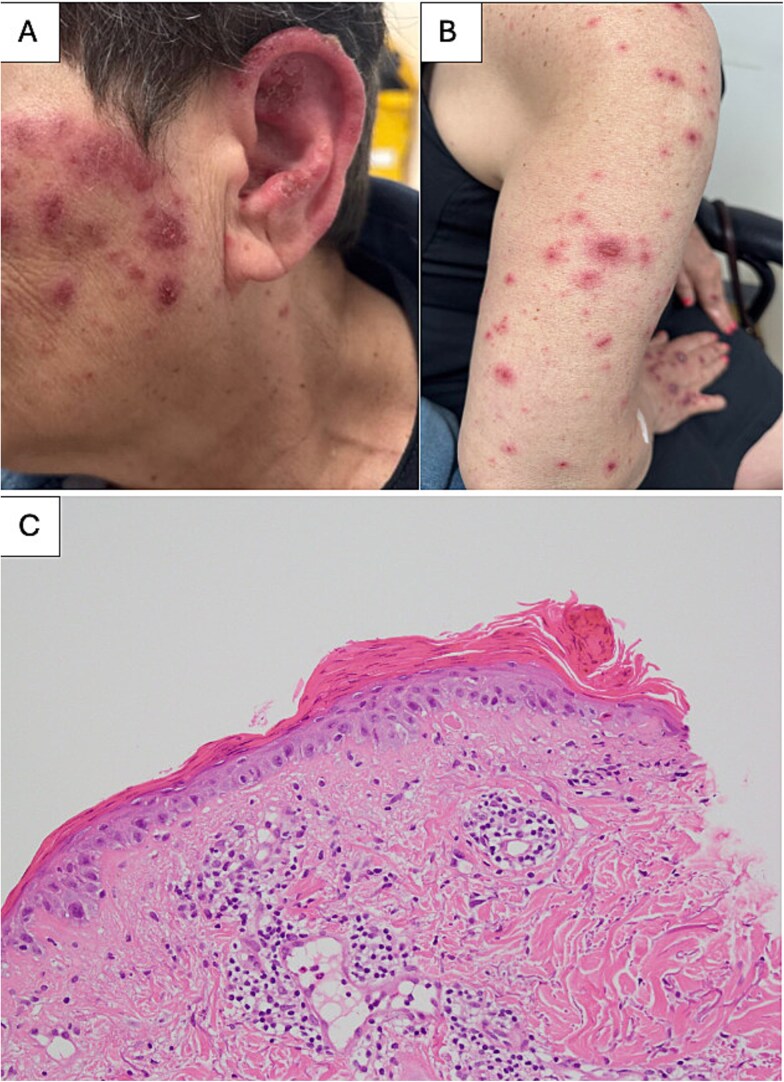
(A and B) Photograph of widespread non-tender erythematous, scaly, annular and polycyclic rash on left cheek and right posterior arm respectively. (C) Histopathology slide demonstrating parakeratosis, attenuated epidermis, apoptotic basal keratinocytes, basal vacuolar change and spongiosis suggestive of acute epidermal necrosis.

A 70-year-old woman with long-standing seropositive, nodular rheumatoid arthritis was commenced on adalimumab, a tumor necrosis factor- α (TNF-α) inhibitor for polyarthritis despite treatment with methotrexate and leflunomide. Two weeks later, she developed a widespread non-tender erythematous, scaly, annular and polycyclic rash without oral involvement (Fig. 1A and B).

A skin biopsy showed parakeratosis, attenuated epidermis, apoptotic basal keratinocytes, basal vacuolar change and spongiosis suggestive of acute epidermal necrosis (Fig. 1C). Her rash progressed despite withholding adalimumab. A repeat skin biopsy confirmed a diagnosis of erythema multiforme. Further serology was done and the diagnosis of Rowell syndrome was made based on presence of subacute cutaneous lupus erythematosus erythema multiforme-like lesions supported by positive anti-nuclear antibody (ANA) 1:160 speckled pattern, anti-SS-A/Ro and rheumatoid factor(RF). Her skin lesions responded well to systemic and topical steroid therapy, and she was subsequently started on hydroxychloroquine.

Rowell Syndrome (RS) is a disease characterized by both lupus erythematosus (LE) and erythema multiforme (EM)-like lesions in subjects with a characteristic immunological pattern. Rowell et al. first described a diagnostic criteria for RS which includes presence of discoid lupus erythematosus (DLE) and EM-like lesions, positive RF, speckled ANA and anti-Ro/SS-A positivity [1]. The diagnostic criteria for RS remains unclear to this date with different variants of criteria available including by Lee et al. in 1995, Zeitouni et al. in 2000 and Torchia et al. in 2012 [2–4]. Furthermore, the role of histological findings as part of the diagnostic criteria for RS remains unclear.

This case highlights the possibility of developing Rowell syndrome, an erythema multiforme-variant of cutaneous systemic lupus erythematosus following initiation of TNF-α inhibitors.
